# Practical Observations on Injuries of the Head

**Published:** 1842-01

**Authors:** 


### Art. VI.

*Practical Observations on Injuries of the Head.* By William Sharp, f.r.s., Senior Surgeon to the Bradford Infirmary, &c.—*London,* 1841. 8vo, pp. 168.

To the practical surgeon, injuries of the head have at all times proved a favorite study, and the ordinary accidents of life have hitherto afforded him no limited sphere wherein to exercise his skill in regard to them. In many spots of the sister isle this class of injury long ranked (to borrow a word from our author) as *endemic,* occasionally assuming also an epidemic character, and the scars of a broken pate were almost as common as those of the smallpox ; but it is gratifying to be able to state that, of late, one man, and he not of our profession, by wisely removing the predisposing cause, has remarkably checked, nay well-nigh uprooted, the most prevalent species of this uncomfortable affliction of the people. In this country, even had similar attempts proved successful, as we are sorry to think has not been the case ; still the spirit of enterprise and manufacture, prolific in railways, engines, and machinery of all kinds, would have left no room to fear that the surgeon of the present day had need to rest content with a diminished field of observation. We have no fault to find with this railway age in that respect, for in it and by it injuries of the head and other parts are at least as plentiful as heretofore ; though, perhaps, there may be often good reason to complain, as did the worthy Doctor Sitgreaves of the puissant Captain Lawton, that the blow is accompanied with such an ill-calculated degree of brute violence, that seldom a sufficient opportunity is afforded for the display of professional skill and dexterity. But be that as it may, the surgeon is still anxious for further information on the nature and treatment of injuries of the head; and although he possesses already the valuable writings of his countrymen, Pott, Samuel Sharp, Hunter, Hey, Abernethy, Bell, Cooper, Brodie, Liston, &c., he hears hopefully that to the name of Sharp, though not Samuel, is appended something like another "Critical Enquiry into the present state of Surgery" on this subject.

It is our duty to convey the results of this enquiry to those who may not have been able personally to become acquainted with it. And although we must honestly confess that disappointment has attended our perusal, and though the conviction is painfully distinct that the author must not venture, even in hope, into the same honoured place with his namesake; yet as the work is by a man of extensive experience, who professes to have bestowed much care on its production, we hope to be able to extract from it some sound practical information regarding "what ought to be considered the best rules to guide us in the present state of our knowledge," (p. 15:) we feel it also imperative on us to notice certain sins, both of omission and commission, and briefly yet plainly to rectify the one and supply the other.

The subject is naturally divided into Concussion, Compression, and Wounds of the Brain; their causes, nature, and treatment.

CONCUSSION. We most perfectly agree with the author as to the propriety of strictly *defining* the term, so as to obviate the confusion caused by "neglecting to separate cases of concussion, from all in which extravasation or other visible lesion of the brain has occurred." In proof that such confusion does exist, he quotes from a recent paper in a medical journal,* where certainly the writer makes his interpretation of "concussion of the brain" sufficiently extended, insomuch that one is only surprised that he should have omitted porrigo, encysted tumours of the scalp, and one or two other affections of the head fully as much connected with concussion as many of those which he has included under the term. But more mischievous errors than this are to be found in surgical literature. In some recent "Systems of Surgery," the symptoms and characters of concussion and compression are so jumbled together as to render it utterly impossible for the student, however anxious, to make out from *that* source any distinction between the two affections. We readily admit that such distinction is often more easily found in the closet than at the bedside of the patient, still there *is* a distinction, and a tolerably plain one too; and surely a systematic writer on surgery is much to blame, if he neglect to make that apparent to the pupil. As to definition, the author wishes that the "term concussion or commotion of the brain should be restricted to cases of an affection 'of one or many functions, without perceptible change of structure in the cerebral fibre,' or other morbid appearance within the cranium; or, in the words of Benjamin Bell, 'such a derangement of the brain as obstructs its natural and usual exertions; but which does not leave such marks of its existence behind it, as to render it capable of having its real nature ascertained by dissection;'" and these symptoms may be the result of violence either applied to the head itself, or communicated to it from some other portion of the body, as by falls on the breech, feet, or hands. To this definition we do not

* Medical Gazette, June, 1839.

object, but we certainly doubt the propriety of including within it cases of injury done to the head, in which the *symptoms do not appear for some considerable time after the accident.* We hold that immediate occurrence is essential to the existence of concussion; and that those cases in which the patient at the time feels little or no inconvenience, but after some days begins to complain, and goes on from bad to worse, often to a fatal issue, if not actively and judiciously treated, are not examples of concussion. The affection is one of inflammation, more or less modified by circumstances, and comes not within the pale of concussion at all. The patient has escaped this, at least it has not occurred to such an extent as to display itself by the ordinary symptoms, yet sufficient impression has been made on the brain or its membranes to predispose to inflammation of either or both of these tissues. In such circumstances, slight causes suffice to kindle the inordinate action, it may be days or weeks after the original injury; and if it occur in the cerebral tissue, it is likely to prove insidious in its progress, and dangerous in the result. For experience daily tells us how often chronic inflammation of the brain, whether the result of injury or idiopathic, advances to destruction of life, without awakening patient, friends, or medical attendant, to a sense of danger, until all room for treatment or for hope has long gone by. When the author speaks of a case in which "the most alarming symptoms followed a fall, though the head was in no visible way injured, and for two days the patient made no complaint," (p. 25,) he does wrong in naming it concussion. And more especially when he tells us of a boy who "fell down a few steps, was stunned for a short time, but soon seemed well," and who, "*several months after*, began to complain of headach, became feverish," and in whom, "notwithstanding the employment of very active depletive measures, squinting, dilated pupils, and excessively violent convulsions came on, the last fit of which continued three quarters of an hour, and ceased only with life," dissection showing "a ramollissement of the central portions of the brain to a considerable extent," (p. 19;) when he calls this concussion, he most glaringly transgresses his own rule, and confounds concussion with inflammation of the brain. We might as well say that hernia cerebri is synonymous with fracture of the cranium, false aneurism with wound of an artery, or phlebitis with venesection. We accordingly object to the following classification of the cases of concussion:

"The first class: Those cases in which alarming symptoms arise immediately on the injury being received, which either soon terminate in death, or speedily pass off altogether. The second class: Cases of concussion in which a few days will elapse after the accident, and then violent and dangerous symptoms suddenly come on. The third class:—cases in which after an accident has occurred, only slight complaints are made for a long time, and which are generally disregarded, until it is discovered that inflammation has been going on and is probably ending in a fatal effusion of serum, in softening of the brain, or in suppuration." (pp. 29-30.)

To the first only and some cases of the second would we limit the term; the others being not cases of concussion proper, but its distant and not unavoidable results.

At page 33, a case is narrated of a "very stout person" who tumbled out of a gig, and bruised his left hip, arm, and side; "his head had not touched the ground, nor had it been struck or knocked against anything." He "complained very little, and not at all of his head." He proceeded on his journey, and "dined heartily. He afterwards got bled to nearly two pounds, and until he fainted. He returned home in the evening (a distance of eight miles), appearing scarcely at all indisposed." Next day he had no complaint, and attended his counting room as usual. On the third day he had "pain and throbbing in the head for the first time . . . . . . with fits of dizziness and of suffocating flatulence." He was bled, purged, had antiflatulent draughts, &c., and after various alternations recovered. Yet this case is called one of concussion! If abscess over the olecranon had resulted from the bruise by the fall, without any head symptoms whatever, that would have had nearly as good a claim to the title.

In regard to the *symptoms* of concussion, Mr. Sharp adopts the statement of Benjamin Bell, (who, though a voluminous and somewhat ancient authority in surgery, nevertheless sometimes said a good thing, but who is much too often quoted by our author,) that the most material difference between the symptoms produced by concussion and compression is in the breathing and the pulse.

1. "In concussion the symptoms usually make their appearance immediately after the accident, though it not unfrequently happens that a considerable interval passes over before they come on; in compression there is generally a short interval between the accident and the symptoms.

2. In concussion, the symptoms if not so overpowering as soon to terminate in death, either pass away, the first signs of depression being followed by corresponding excitement, and this by complete recovery; or we may have the three stages pointed out by Mr. Abernethy, first of insensibility, second of restored animation, and third of inflammatory action. In compression, the symptoms if not relieved by active depletion, and other means, become aggravated, and death ensues either very soon, or after inflammation, and frequently also suppuration have supervened.

3. In concussion, the pulse is generally not so slow, labouring, and intermittent as in compression.

4. In concussion, bleeding sinks the pulse and the patient; in compression the pulse rises and relief is afforded.

5. In concussion, there is commonly more insensibility, followed by delirium, than in compression.

6. In concussion, perhaps vomiting is more frequent than in compression, though it often occurs in the latter.

7. In concussion, there is less frequently coma, convulsions, and paralysis, than in compression.

8. The state of the pupil is an uncertain sign; it is often dilated and insensible to light, and often contracted in both concussion and compression.

9. Sometimes at the moment of the accident, the patient has a sensation of flashes of light before his eyes, or of noise in his ears, and is as if intoxicated; sometimes the first symptoms are followed by furious and raving delirium, which, however, soon subsides; and sometimes individual senses remain affected for a very long time after the injury, as the sight, smell, taste, or hearing." (pp. 48-50.)

Touching No. 1, we have already stated wherein we disagree with Mr. Sharp. As to No. 5, the wording is sufficiently obscure, and the apparent meaning moreover seems incorrect. In other respects, the statement is valuable as being the result of his own observation; and the more so as it accords with and consequently confirms what has been already laid down upon this subject by our best surgical authorities.

In *treating* concussion, surely no educated surgeon would now think of trephining the skull. In case, however, any one should be afflicted with a cacoethes operandi in that way, he will find the reasons for withholding his hand, clearly and succinctly stated, as follows:

"There is no depressed bone to elevate; no extravasated blood to evacuate; no tension to relieve; nor can inflammation be prevented by adding to the shock which has produced the concussion, the further injury of removing a portion of the cranium, and exposing the membranes of the brain." (pp. 52-3.)

Of the statement which follows, however, to the effect that in concussion the surgeon "will aim at being of service to his patient chiefly by *general* treatment," we disapprove, as tending to mislead the young practitioner. There is *much local* treatment. There is shaving of the head, elevation of it, and constant application of cold; leeches ultimately, or arteriotomy, if reaction has not only been established, but threatens to run into excess, and where the patient by look, gesture, movement of the hands, or muttered speech, indicates that there is pain there. But above all, there is the most important part of the local treatment, namely, complete rest of the injured organ, so far as that is attainable. In the case of a similar injury inflicted on the chest, a broad bandage is applied tightly to restrain the involuntary movements in respiration, if cough is present suitable medicines are given to prevent it, the patient is interdicted from speaking, blowing his nose, &c., the object being to limit the parts simply to what duty by them is essential in the general business of vital function. And so it ought to be in regard to the head. Not only must all general excitement be eschewed, but more especially must all sources of excitement, in which the brain is likely to be more immediately concerned, be carefully avoided. The brain quietly carries on the business which is allotted to it as its ordinary and everyday work, and our object is to see that it does nothing more. This is local treatment surely, and one of a most important kind.

We have much pleasure in stating how cordially we agree with our author in reprehending an absurd practice, unfortunately too common both in and out of our profession, of injudiciously administering stimuli during the first stages of concussion. On this point nothing can be better than the words of Sir Benjamin Brodie, as quoted by our author:

"There are, indeed, sufficient reasons why we should regard that condition of the system which approaches to syncope, as being in the great majority of instances in which it exists, conducive to the patient's welfare, and why we should wish to prolong, rather than to abridge, the period of its duration; the same blow which gives rise to symptoms of concussion, frequently occasions the rupture of some small vessels within the cranium. The same state of the system which produces an enfeebled action of the heart, is calculated to prevent the ruptured vessels from pouring out their contents; and the longer it continues, the less is the danger of internal hemorrhage. If we artificially excite the action of the heart, by the exhibition of wine and ammonia, we are in danger of inducing symptoms of pressure on the brain. If on the contrary we watch the gradual restoration of the pulse, and at the proper moment take from the arm a sufficient quantity of blood, to prevent the heart resuming its wonted action, it is probable that we may often succeed in checking or arresting an extravasation of blood on the surface of the brain, or among its membranes, which might otherwise prove fatal. There is also the following very important circumstance which is not to be overlooked in this part of the enquiry. A state of depression is followed by a state of excitement; as the patient recovers from the former, the pulse with respect to fulness and strength, becomes raised above the natural standard, and it is evident that this affords an additional argument in favour of the practice which is here recommended." (pp. 54-5.)

There is another error, however, fully as common as the preceding, and equally dangerous, if not more so; the very opposite procedure; instantly bleeding, or attempting to bleed, in the first stage of concussion; in other words assisting the injury to put a stop to the heart's action, and so to terminate existence. To this Mr. Sharp has not alluded; and we are thence led to hope that in his part of the country such malpractice is not very prominent. Having seen not a little of it ourselves, however, in unavailing sorrow, we here take the liberty to supply the omission. It was, and still is, too much the custom to bleed, so as to appear to be doing something active in treatment, not only when the proceding has seemed to the ignorant practitioner likely to do no harm, but also when a wiser head could have told that it must do irremediable mischief. The good fortune of the first phlebotomist, Podalirius, seems to have produced in his successors an unfortunate attachment to the operation, which has been communicated from generation to generation, and from which our day is not yet wholly exempt. What can be worse than to find a patient who has just sustained a severe concussion—pulseless, and with the powers of the system all but extinguished—robbed by an ignorant or reckless man, unfortunately called to his aid, of those very powers of which he is at the time most in need, and without which the depression must sooner or later pass into extinction, or death? But, in the words of our author,

"On reaction taking place, blood may be safely taken from the system, and this abstraction of blood should be continued and repeated, in proportion to the strength of the patient, as long as the pain or excitement continue." (p. 56.)

*Purgatives*, it is well known, have a marked and rapid effect on the brain, diminishing the amount of its circulating fluid, and consequently depressing excitement, whether natural or accidental, of that organ. This class of remedies are thus invaluable in both warding off and moderating inflammation either of the brain or its membranes, and ought to go hand in hand with bloodletting in the treatment of those cases of concussion in which reaction threatens to be, or has become excessive. Yet Mr. Sharp but cursorily alludes to them. *Opium*, we also know, has an opposite effect, producing a determination of blood to the head, more especially accumulating the venous portion; and therefore its use is inadmissible, except under peculiar circumstances.

"Opium is objectionable, unless bleeding has been carried so far as to give rise to restlessness, and other unpleasant symptoms, when a small quantity may sometimes be found beneficial; but its effects should be carefully watched, and if not beneficial, its use should be discontinued. We are advised to combine it with antimony. (p. 57.) . . . . "A question of importance is to determine the period *when opium may be advantageously given*. In some cases there is a fortunate moment in which, if the practitioner know how to seize it, opium will act almost like a charm; whereas at another time, in the same case, it would do much mischief." (p. 65.)

This is all that is stated in regard to opium, and the statement is surely far too vague. The author should have pointed out why this drug is in general inadmissible, and *why and when* it becomes appropriate. The former omission we have already endeavoured to supply. And we now add briefly, that "the fortunate moment in which opium will sometimes operate as a charm," is when in consequence of full or repeated bleeding, the system has been placed in that state of *nervous reaction*, marked by the peculiar jerking pulse and other symptoms well known to the practical surgeon and physician. Yet, even then, the drug must be given with a sparing hand and watchful eye, lest the untoward effects already stated should supervene.

As to the treatment of those insidious and dangerous cases, formerly alluded to, in which the head symptoms occur at some considerable distance from the period of the injury, we have the following judicious observations:

"Nothing alarming occurs for a long interval, during which the patient is not well, but he complains of little; a slight headach, occasional giddiness, sometimes a disposition to vomit, a somewhat quickened circulation, perhaps an inflamed eye, languor of mind and lassitude of body, loss of appetite and of sleep, a skin rather hotter and a tongue rather drier than natural. Any of these symptoms a superficial observer will hope to remedy in a few days; but they continue, all remedies unexpectedly fail, one disappointment is followed by another, until the practitioner, as well as the friends of the patient, are suddenly awoke as it were, and alarmed by the too surely fatal symptoms of *pressure*. Let the surgeon then be on his guard; let him anticipate, and if possible ward off the fatal blow: in these cases nothing is more dangerous than a sense of security; the slightness of the symptoms must not be considered, but their continuance, and no treatment must be thought satisfactory, but that which entirely removes every complaint.

"With regard to the particular measures to be adopted, the surgeon who is alive to the true nature of the case, will not find his difficulty arise in their selection, but in persuading his patient to submit to their adoption; here again, my own painful experience is confirmed by that of Pott: 'I am very sensible,' he says, 'that it will in general be found very difficult to persuade a person, who has had what may be called only a knock on the pate, to submit to such discipline, especially if he finds himself tolerably well. He will be inclined to think that the surgeon is either unnecessarily apprehensive, or guilty of a much worse fault.'" (pp. 62-3.)

And at another part of the volume, an instructive case is detailed, which we shall transcribe, *in terrorem*; as it affords an excellent example how great and irretrievable mischief must ever result from careless and inaccurate diagnosis in such matters:

"1814, April 19th. Mr. John Sharp was riding, and by some means his horse got from a trot to a gallop, and having lost his command, to avoid passing through the toll-gate at that speed, he quitted his horse, and by the fall bruised his knee a little, and also his head; neither of which bruises caused the least alarm, as the skin was but just broken. In the course of four or five days, some inflammation of one eye took place, but this was considered only the effect of cold; but on the *eighth* day from the fall, a violent headach came on, and on the tenth he was cupped, which removed the pain for a few days; it then returned, and a blister was applied between the shoulders; this again gave relief for a time, but the pain returned and the fever continued. A physician was called in, who considered the accident of the fall from the horse of no consequence whatever, but attributed the complaint to the bad state of the constitution, as in his opinion no symptoms from the effects of the fall appeared, (though he had been previously a remarkably strong healthy youth;) a bracing, tonic plan was adopted, accompanied with good living; bark, jellies, &c. In about a fortnight he was pronounced out of danger, although the headach continued; and on the 14th of June, he was sent into the country for change of air; although the pain in the head was getting worse. He became much worse immediately after the journey. On the 23d he took to his bed. On the 24th a stupor came upon him, which deprived him of sense. On the 25th, his head was shaved, and a large blister applied, which discharged copiously, and in some degree diminished the stupor, but it was very far indeed from being removed; he continued insensible and helpless, and died on the 1st of July." (pp. 41-3.)

It seems at first startling to assert, that the severer forms of concussion are more dangerous than extensive fractures, and therefore ought to be more attentively watched in the treatment. The reason is obvious, however, when we reflect, that the extent of commotion in the brain by the injury, depends greatly on the continuity of the calvarium, through which the concussion is communicated to the contents; and that when once that continuity has been broken, the extent of violence to the brain is proportionally diminished, except in the case of direct injury by pressure or puncture. To confirm this practical fact, Mr. Sharp adds the weight of his experience.

As to the *diagnosis* of injuries of the head, we have the following observation: "The nature of the cause which has produced the injury will often assist us in distinguishing it; for instance, a violent shake of the whole body is very likely to produce concussion but not compression of the brain; on the contrary, a smart blow with a poker or a stone will most probably give rise to compression." (pp. 71-2.)

With the general proposition we agree, but think the illustrations unhappy. "A violent shake of the whole body" is as likely to produce compression as concussion. How often, for example, do we not see the worst of all this class of injuries, fracture of the base of the brain with hopeless extravasation and compression, occurring in consequence of violent succussion of the body by falling from a height on the feet or breech? A blow by a poker, if violent, is doubtless very likely to occasion a depressed fracture with compression, and the stroke of a stone may produce a fracture either punctured or depressed, according as the implement is angular or rounded; yet we see no reason why either, lightly administered, may not occasion simple concussion. In the same page (72) diagnosis by the direct cause is carried still further; and we are told that "a collier, while working in the pit, had a quantity of the roof over him fall on the back of his head, while he was engaged in pushing before him a small waggon; and being very much bent forward, his head was forced violently against a part of the waggon, so as to fracture the frontal bone. In this case concussion was caused by the earth falling upon the occiput, and compression from fracture with depressed bone, by the iron against which the forehead was struck." Really we suspect that our author will find very considerable difficulty in thus limiting earth and iron to separate and distinct offices, when combined to inflict lethal injuries.

Compression. Compressed brain is considered under three heads: Compression from extravasated blood, from depressed bone, and from abscess and effusion. Under the first of these, nothing new is stated; but some points both of theory and practice, of an obviously dangerous tendency, require our attention. That "sometimes blood will be extravasated

(on the brain) and produce no symptoms at all" (p. 80), is scarcely credible, unless the effusion be not only most gradual but of most limited extent. But to this we do not so much object as to what follows. After having briefly, too briefly and generally, alluded to the advantages of bloodletting, and the rest of the treatment suited to prevent increase of the pressure by further extravasation of blood, he adds, "When these means, actively employed, fail to afford relief, and the patient is evidently in a desperate condition, there is still one remedy remaining, namely, *the trephine.*" (pp. 83-4.) And lest there should be any doubt about his opinion, we find this rule laid down at page 113, that "when we are called to a patient labouring under the symptoms of compression of the brain, with a contusion, or wound on the head, and there is no fracture, we are to perforate the bone in the hope of finding either extravasated blood," &c. Here we are completely at variance with our author. We are aware that it is still a point not altogether settled, whether the trephine ought to be employed in cases of compression by extravasated blood; but we hold that it ought not, as a general rule, and for the following reasons: 1. It is impossible to ascertain at what part of the brain the extravasation is situated; whether at the point of injury, or opposite, at the site of the contre coup; or at some other part of the brain, on the surface, at the base, in the ventricles, or in its substance. And surely, in such uncertainty, a surgeon is not warranted in boring here and there for effused blood, as an engineer would for water or coals. 2. It is impossible to ascertain where the extravasation is situated, in regard to the cranial contents in general, whether on the dura mater, on the arachnoid, on the pia mater, or on the brain. Let Mr. Sharp himself speak to this. "Examination after death teaches us that blood may be poured out and coagulated in any of these places (already enumerated); and when the accidents are very severe, in several, or even all of them together. *But in the present state of our knowledge they cannot be distinguished from each other by the symptoms they give rise to during life.*" (p. 75). So that, even granting that we were able to say (which we are not) that opposite a particular part of the skull extravasation of blood has taken place, we have no surety but that, in order to reach that extravasation, it may be necessary to perforate not only the skull, but one and all of the membranes of the brain; and the propriety of such a measure may well form the subject of grave consideration. 3. Supposing the blood has been reached, the probability is that we shall be unable to remove it. If fluid, it will escape spontaneously, as do pus and serum in similar circumstances; but in the great majority of cases it will be found coagulated, in the shape of a flat mass; and to remove this would require a disturbance of the brain and its envelopes, infinitely more destructive than the original mischief. At page 80 we find the following: "On the sixth day he was decidedly suffering from compression, and the trephine was applied on the injured part of the parietal bone: a coagulum of blood was found, but it was under the dura mater. He died on the eighth day." This is carelessly enough stated, yet the following inferences are plainly deducible: that the coagulum was either not reached, or if it was, it was by perforation of the dura mater, and the result proved fatal in a few days. And equally unfortunate will be found the terminations of an overwhelming majority of operations performed under similar circumstances.

4. A man with a flat coagulum compressing his brain more or less injuriously, is not improved, as to his chance of recovery, by having that compressing body either imperfectly removed, or not removed at all, while the addition is made of an extensive incision in the cranial coverings, or hole in the cranium itself, a wound of one and all of the membranes of the brain, and more or less violence by manipulation done both to these and to the organ itself. And therefore, we say again, let such cases remain as they are, contenting ourselves with assisting nature in *her* mode of cure, by absorption and mutual accommodation, and certainly refraining from the performance of an operation, which can do no good to the patient, and whose failure will certainly entail discredit upon surgery. The only circumstances in which we can suppose the operation at all warrantable, are when the blow has been received over the course of the middle meningeal artery; and when, consequently, the probability is that the blood is seated there, and between the bone and dura mater. In such a case, the diagnostic symptoms being very plain, the surgeon may be warranted in removing a portion of the skull; but, failing at this point of the search, he must not go further and penetrate the membrane. We are aware that there are a few successful cases on record in which extravasated blood has been removed by the agency of the trephine; for example, that related by Mr. Abernethy, in which the old woman, previously insensible, jumped up in bed, and very appropriately demanded "what the devil they were all about;" but in such cases the blood had fortunately remained fluid, and they are plainly to be regarded as mere exceptions to the general rule.

On the subject of *compression by depressed bone*, we have a plain and good statement as to the folly and danger of officious meddlesome surgery, and of the means whereby the practitioner is to distinguish between bruise of the scalp and depressed fracture of the skull:

"It is very necessary to be on our guard against the deceptive appearances produced by a blow on the head, causing effusion into the cellular membrane, and under the tendon of the occipito-frontalis muscle, without injuring the bone. This gives rise to appearances exactly similar to those of simple fracture with depression, that is, a soft hollow centre, surrounded by a hard ridge, communicating such impressions to the inexperienced eye and finger as will certainly deceive them. A careful examination will show, First, that the ridge is above the level of the adjacent sound bone; secondly, that the hollow is not below it; and thirdly, by pressing the finger rather firmly on the ridge, for a few moments, it will produce a pit or depression, and then the bone may be felt. In a short time the effusion is absorbed, and these deceptive symptoms disappear. With this exception, fractures of the skull are, in general, easily detected. Secondly, in a doubtful case we are not justified in making a wound, where there is not one already, merely to ascertain whether there be a fracture or not; unless urgent symptoms of pressure call for active interference to remove it. Thirdly, where there is a wound and fracture, we ought to be very careful in our use of the probe, lest we push it, or a fragment of bone by it, so as to increase the injury done to the brain. Fourthly, a fissure, or slight fracture of the cranium without depression, is not in itself dangerous, and does not require any peculiar treatment; the caution most needed, in such a case, is to avoid officiousness, and to prevent inflammation." (pp. 89-91.)

Several cases are adduced in corroboration of the opinion, already pretty well established, that operative interference is unnecessary in cases of depressed fracture, unless symptoms of compression are not only present but of an urgent character. But sufficient attention is not paid to one of the most formidable injuries to which the head is liable, namely, fracture causing a disruption of the inner table far exceeding that of the external, usually termed "punctured fracture," as being ordinarily produced by sharp-pointed weapons, and demanding the instant application of the trephine, whether untoward symptoms have as yet occurred or not. This important class of injury is little more than alluded to; the only instance adduced is from Cooper's Surgical Dictionary, and is rather an unfortunate example, as being likely to lead to a false notion of the usual mode of production, the injury having in this case been occasioned by a musket-bullet. We shall briefly state the nature and treatment of this kind of fracture, by an apposite quotation from the article "Surgery" in the Encyclopædia Britannica, 7th edition, written, we believe, by Mr. Miller, Surgeon to the Edinburgh Royal Infirmary:

"The punctured or star-like fracture is occasioned by a sharp body striking the head with considerable force. The integuments are divided, and the surface of the bone presents an appearance of injury somewhat resembling what is often seen in ice when struck in a similar way. But this is a very slight extent of damage compared with what the inner table suffers, when the puncturing weapon has passed through the diplöe, as is usually the case. The inner table, being by much the more vitreous, is shivered into numerous spiculæ, which being driven inwards by the force of the blow, perforate, or at all events grievously irritate, the coverings of the brain, producing inflammatory action, soon affecting that important viscus, and, if not arrested, proving speedily fatal. After the infliction of such an accident, therefore, even should the patient be at the time so little affected as to walk to the surgeon to have his wound dressed, the trephine should be immediately applied to the punctured point, in order that a portion of the bone may be removed, sufficient to allow extraction of all the displaced portions of the internal plate. In no other way can we avert intense inflammatory action from the wounded dura mater, extending in all probability to the brain and its more immediate investments. Even should the practitioner be in some degree successful in moderating the more immediate mischief by antiphlogistics alone, the necessity for trephining will still remain, on account of abscess under the bone, occasioned and kept up by the spiculæ; the matter must be evacuated, and the cause of its formation must be removed. It is surely infinitely better, therefore, to operate in the first instance, and so avert all such calamities." (p. 718.)

For cases amply illustrative both of the benefit of the operation and the danger of delay in such cases, we would refer our readers to Mr. Liston's Practical Surgery, 2d edition, p. 47 et seq.

Under "*compression of the brain from suppuration*," we find the following theory of the fluid's formation: "Matter forms under the tendon of the occipito-frontalis muscle; the pericranium becomes inflamed and detached from the skull; *the inflammation is communicated through the bone to the dura mater;* that membrane is separated from the bone; pus is formed on its surface; and the patient dies, labouring under the symptoms of compression of the brain." (p. 114.) Verily this is contiguous sympathy with a vengeance; and rather a clumsy chain of reasoning in the present day. The propriety of operation in such cases is well stated, however, as also the means of diagnosis; but we cannot agree that the "soft circumscribed puffy tumour" is "a *sure* indication of the detachment of the pericranium from the outer, or of the dura mater from the inner surface of the bone." (p. 115.) The coexistence is not invariable.

And though the surgeon is not only warranted but required to use the trephine, when head symptoms are accompanied by this peculiar condition of the scalp, he must be prepared for occasional disappointment as to the object of his search. Yet let him not be easily baulked in his pursuit; if the diagnostic symptoms are very marked, let him apply the crown of the instrument again and even again. For it is to be remembered that, differently from what obtains in regard to compressing blood, if compressing pus be not evacuated, it must inevitably prove fatal; that this abscess, unlike those in other situations, can hardly make its way to the surface for discharge; and that, though such a fortunate circumstance has occasionally taken place, as by absorption and discharge into the ear, still it would be worse than folly to treat a case with the vain expectation of a similar godsend.

As to the *operation of trephining*, we pass by a condensed history of the varying principles by which it has been guided, from the time of Hippocrates downwards, and give the result of the author's own experience:

"1. Not to trepan in cases of concussion. 2. Nor in cases of compression of the brain, unless there is very good reason to expect extravasated blood immediately under the bone; fracture of the internal table; or pus, indicated by the puffy tumour, or detached pericranium. 3. Nor in children where there is depression only, without fracture. 4. Nor in simple fissures or fractures with slight depression, but without alarming symptoms. 5. Nor even in compound fractures without depression or symptoms.

"And on the other hand,—

"1. To perforate the cranium when extravasated blood or matter may be fairly expected to be found underneath; and to raise the depressed bone, in all cases of depression, whether simple or compound, where there are serious symptoms of pressure. But it still remains an unsettled point whether, 2, A simple fracture with extensive or deep depression, but without symptoms of pressure, and, 3, A compound fracture with depression, but also without symptoms, should be trepanned or not." (pp. 137-8.)

For the reasons already assigned, we object stoutly to the operation undertaken on account of extravasated blood. Nor do we agree that the last-mentioned point is unsettled; but, on the contrary, hold that all true surgeons are bound not to interfere, whatever be the state of the fractured bone, unless urgent "symptoms of pressure" have arisen (and unless loose fragments can be simply lifted away without violence), except in the case of punctured fracture, as formerly stated. Mr. Sharp tells us that he "inclines" to this rule of treatment, but we think he ought to have done more in its favour.

In regard to the performance of the operation, Mr. Sharp very properly opposes the vulgar prejudice against trephining in the immediate vicinity of the longitudinal sinus, meningeal artery, sutures, &c.; there is no risk of any accident thereby which may not be readily obviated; yet it is well to avoid the first two situations, unless circumstances compel us to do otherwise. His proposal to employ "a small chisel (such as we use in necrosis of the tibia,") as being "more expeditious" than the trephine or Hey's saw, is not likely to meet with approbation. There is no operation, in the wide field of surgery, in which speed is less demanded than in this. In most cases the patient is more or less insensible to pain; and in all he suffers comparatively little during perforation of the bone. Surely, then, a clumsy, unsurgical, and withal dangerous instrument ought not to supersede those which are not only safe but "expeditious" enough. He properly observes that the considerable loss of blood which usually takes place during the operation is of advantage to the patient, as prophylactic; yet we would warn the profession against carrying this maxim too far; inasmuch as we have seen a temporal branch spouting during the application of the trephine, ceasing ere the conclusion of the operation, receiving no ligature, and, on reaction having commenced, again bleeding to such an extent as to prove highly injurious.

Wounds of the Brain. In his observations on wounds of the brain, our author is far from being prolific, but we thank him for the following:

"The question of removing fragments of bone or of foreign bodies can only be entertained when those fragments are so loose as to be extracted without doing further injury to the brain; the result of my own practice is in favour of their being removed; while the result of my reading is, on the contrary, in favour of their being left. There can be no doubt of the propriety of leaving, unmolested, all cases where the depressed bone, or the foreign body, a bullet for example, is so circumstanced that, to extract it, the brain must suffer still further laceration. It will perhaps appear, prima facie, that I have pursued an opposite course of practice in these cases to that recommended in the preceding chapter; but when it is considered how different the cases are—that in the former, the brain is only pressed upon, and that without its giving evidence of suffering, while in the latter, it has a sharp jagged edge of bone continuously puncturing it; it will not then appear surprising if a different mode of treatment be thought necessary." (pp. 153-4.)

The following practical observation, with illustrative case, is also of much importance, and not sufficiently attended to in general:

"Where the patient recovers with loss of some portion of the bony covering of the brain, it is highly necessary that he should be provided with a metallic covering for the part which has lost its bony protection, before the surgeon takes leave of him, and before he is allowed to go much out of doors, or to engage in any active occupation or amusement. The necessity for this precaution is rendered painfully evident by the following case:

"1828. A boy, about eight years old, received a severe blow on the left parietal bone by the fall of a coal, while employed at the bottom of a pit. I found a large wound, a comminuted fracture of the bone, and a laceration of the dura mater; he was in a state of extreme exhaustion, and in fact, it seemed a hopeless case. I removed the splintered bone, and lightly dressed the wound, and to my great surprise and delight, the boy recovered. He got out of doors, before I was aware of it, and, boylike, engaged in full play, with some of his companions; he was struck by a stone, on the very spot where the bone had been removed, and he died in a few days. I mention this painful circumstance, believing that it will produce as strong an impression on the minds of other surgeons as it has upon my own; and in the hope that we may be able so to put our patients on their guard, that a similar catastrophe may, in future, be prevented. A small plate of tin, or other metal, may be made to fit over the part without difficulty. I am at present attending a case of this kind, in which, in consequence of a blow from a stone, received nearly three years ago, several large portions of the parietal and temporal bones have exfoliated, and have been extracted, by which the dura mater is exposed to a great extent; it is now protected by a large metallic plate which is constantly worn." (pp. 153-6.)

We have now come to our last quotation:

"With regard to the imperfections in the execution of my work, which will doubtless be discovered by those who have leisure to peruse it critically, it may suffice to remind them, that it has been written in the midst of the distractions and interruptions of daily practice; which, indeed, would have discouraged me altogether, had I not wished to put upon record the practical results of a great many observations. 'Feci quod potui, non ut volui.'" (p. 160.)

This is always the way. A book is got up and thrown off. The parent is pleased with and fond of his offspring; but not certain of its being as tenderly entertained by the rude public as he could wish, he hopes to smooth its way by apologetic allusions to "want of leisure," "distractions of daily practice," &c., and quotes Latin to protest that he has done the best he was able. But we have long since determined to disregard all such ill-grounded appeals to our critical forbearance, and certainly do not see any necessity of foregoing our custom in the present instance. Mr. Sharp has as good reason for publishing his book as the majority of authors of the present day have. Although not very cleverly written, and containing too much of other people's and too little of his own, we believe its appearance will be useful to the profession at the present moment. We think, indeed, that a surgeon who has practised where injuries of the head are "endemic," and who "has devoted a large share of his attention to the subject during upwards of twenty years," ought to have produced a work of higher value than the present. Accordingly, while we have most willingly bestowed our praise on those portions which seemed to deserve it, we have not hesitated to blame those which appeared to us not only erroneous but fraught with danger to the inexperienced reader. We trust that Mr. Sharp will take our observations in good part; and, should a second edition be attained, we hope that he will therein quote less from previous authors, and more from his own experience; and when he has studiously reconsidered the nature and treatment of the various injuries of the head, with their probable consequences, both generally and in detail, doubtless his book will then prove more interesting to the practical surgeon, safer to the student, more blameless in the eyes of the reviewer, and more worthy of himself and his opportunities.

---

